# Fatty acid composition in breastfeeding and school performance in children aged 12 years

**DOI:** 10.1007/s00394-015-1030-y

**Published:** 2015-09-07

**Authors:** Geertje W. Dalmeijer, Alet H. Wijga, Ulrike Gehring, Carry M. Renders, Gerard H. Koppelman, Henriette A. Smit, Lenie van Rossem

**Affiliations:** 1 Julius Center for Health Sciences and Primary Care, University Medical Center Utrecht, STR 6.131, PO Box 85500, 3508GA Utrecht, The Netherlands; 2 National Institute for Public Health and the Environment (RIVM), Bilthoven, The Netherlands; 3 Institute for Risk Assessment Sciences, Utrecht University, Utrecht, The Netherlands; 4 Department of Health Sciences, Faculty of Earth and Life Sciences, and EMGO Institute for Health and Care Research, VU University Amsterdam, Amsterdam, The Netherlands; 5 Department of Pediatric Pulmonology and Pediatric Allergology, University Medical Center Groningen, Groningen Research Institute for Asthma and COPD, and Beatrix Children’s Hospital, University of Groningen, Groningen, The Netherlands

**Keywords:** Breastfeeding, Fatty acid composition, School performance, Birth cohort

## Abstract

**Purpose:**

Breastfeeding has been associated with improved cognition. It remains unclear whether long-chain polyunsaturated fatty acids (LC-PUFAs) play a role in this association. We assessed the association between LC-PUFA concentrations in infant feeding and school performance at age 12.

**Methods:**

Within a population-based birth cohort, we compared school performance of 277 non-breastfed children and 157 children who had fatty acid composition of their mothers’ breast milk measured. Two indicators of school performance were: (1) the score on a standardized achievement test and (2) the teacher’s advice regarding a child’s potential performance level in secondary education. Linear regression and multinomial logistic regression analyses were performed to assess the independent association between LC-PUFA content of breast milk and school performance.

**Results:**

Girls, who received breast milk with a relative high content (above the median) of docosahexaenoic acid (DHA), had a higher Cito-test score (*β* = 2.96 points, 95 % CI 0.24; 5.69) than non-breastfed girls. Among the breastfed girls, each percentage point of higher content of total n-3 LC-PUFA (*β* = 4.55, 95 % CI 0.43; 8.66) and DHA (*β* = 7.09, 95 % CI 0.9; 13.3) was associated with a higher Cito-test score. The association between LC-PUFA content and teacher school advice showed a similar pattern. There was no association between LC-PUFA content and school performance in boys.

**Conclusion:**

Although a large part of the association between infant milk feeding and cognition seems to be explained by sociodemographic and lifestyle-related factors, a relative high content of n-3 PUFAs, especially DHA, in breast milk is associated with better school performance in 12-year-old girls but not in boys.

**Electronic supplementary material:**

The online version of this article (doi:10.1007/s00394-015-1030-y) contains supplementary material, which is available to authorized users.

## Introduction

Consistent differences in cognitive performance between breastfed and infant formula-fed infants have been reported [1].

In contrast to the content of infant formula feeding at least up to the end of the twentieth century, breast milk contains long-chain (LC) polyunsaturated fatty acids (PUFAs), which are important structural components of the brain. Hence, it has been hypothesized that the beneficial effect of breastfeeding on cognitive performance may be the result of a long-term effect of LC-PUFAs.

Some observational studies have reported that consumption of oily fish, which is known to be rich in docosahexaenoic acid (DHA), an essential n-3 LC-PUFA, during pregnancy is positively associated with improved cognitive performance of the child [2–5]. However, results of randomized controlled trials of maternal supplementation with n-3 LC-PUFAs during pregnancy and lactation, compared with a control supplementation, have been inconsistent [6]. This is probably due to the relatively small sample sizes (*n* < 100) of the trials and, consequently, their limited statistical power to detect small differences. In addition, in these trials, there is no information about the actual effect of n-3 LC-PUFAs supplementation on the fatty acid composition in breast milk. Another way to investigate the effect of n-3 LC-PUFAs on cognitive performance is to enrich infant formulas with LC-PUFAs. Meta-analyses of randomized clinical trials of infant formulas supplemented with LC-PUFAs did not demonstrate a clear or consistent benefit to cognitive performance by supplementing the formulas with LC-PUFAs [7, 8]. A recent review suggested that sample size, genetic polymorphisms, sex, source of supplement, dose, timing of supplementation, duration of supplementation, compliance with treatment and the selection of the test of assessment of cognitive performance outcomes may be the factors responsible for these inconsistent results [9].

In contrast, two observational studies suggest that higher levels of n-3 LC-PUFAs, directly measured in breast milk, and higher ratios between n-3 and n-6 PUFAs in colostrum, play a beneficial role in cognitive performance measured at 14 months and 6.5 years of age [10, 11]. We would like to proceed in line with these two studies and, with our observational birth cohort study, investigate the association between LC-PUFA composition of infant feeding and cognitive performance, measured as school performance, at 12 years of age.

Due to the possible differences in the metabolic capacity to synthesize n-3 LC-PUFAs [12] based on sex, we will separate the analyses for boys and girls in our study. Furthermore, in contrast to the previous studies, in our study, children who were never breastfed served as reference, because at the time when the children were born (1996–1997), infant formulas did not contain n-3 LC-PUFAs in the Netherlands [13], and therefore, this reference group has never received n-3 LC-PUFAs in their first months of life.

## Subjects and methods

### Study design and setting

The current study was embedded in the Prevention and Incidence of Asthma and Mite Allergy (PIAMA) Study, a population-based Dutch birth cohort study. Details of this study are described elsewhere [14]. In short, pregnant women were recruited from the general population during their first antenatal visit. Their children were born in 1996–1997 and have been followed up to the age of 12 years. The study protocol was approved by the medical ethics committees of the participating institutes, and all parents gave written informed consent. In a subsample of the mothers, breast milk samples were collected around the child’s age of 3 months. At the age of 12 years, information on school performance was obtained. For this observational study, we identified two subgroups from among the children with known school performance: children who were never breastfed (*n* = 277) and children from a subsample of mothers who collected a breast milk sample for fatty acid analyses (*n* = 157).

### Fatty acid composition of breast milk

In the current study, children who were never breastfed served as reference, because at the time the children were born (1996–1997), infant formulas did not contain n-3 LC-PUFAs in the Netherlands [13], and therefore, this reference group had never received DHA and EPA in their first months of life.

Data on breastfeeding initiation were assessed by a questionnaire at age 3 months. We collected information on fatty acid composition of the breast milk from the group of breastfed children.

The fatty acid composition of the breast milk was determined by gas–liquid chromatography, according to Foreman-van Drongelen et al. [15]. The majority of the breast milk samples (77 %) were obtained between 11 and 18 weeks after delivery. Further details of breast milk collection and analysis have been reported elsewhere [16]. Fatty acids were measured as a weight percentage (wt%) of the total fat content. Fatty acids of main interest in relation to cognitive functioning were DHA (22:6[n-3]), EPA (20:5[n-3]), AA (20:4[n-6]), the sum of n-3 LC-PUFAs (≥20), DHA/AA ratio and EPA/AA ratio.

### School performance

The parents received a questionnaire when the children were ~12 years old with which information concerning school performance was collected. As indicator for school performance in this study, we used test results of a standardized school achievement test, the Cito test and teacher’s advice for the level of secondary school. In the Netherlands, secondary education starts at age 12 and consists of several levels which prepare children for vocational education, higher professional education or university. In order to assign children to the most appropriate secondary education levels, first of all, an academic achievement test is administered at the end of their primary education at age 11–12 years. The most commonly used test for this purpose is the (Cito) test which is developed by the National Institute for Educational Measurements (Cito, Centraal Instituut voor Toetsontwikkeling) [17]. The test includes questions concerning spelling, math, study skills and world studies. Together the performance scales result in a standardized score between 501 and 550. On average, a pre-vocational secondary education is recommended to children with a Cito-test score between 501 and 536, a senior general secondary education is recommended to children with a score ranging from 537 to 544, and a pre-university education is recommended to children with a score between 544 and 550 [17].

Another way to measure school performance is the teacher’s school-level advice. The teacher bases his advice on the Cito-test score as well as the entire school career, skills and characteristics of the child. It is common that children receive the advice to participate in a combination class of two education levels (e.g., combination of senior general and pre-university education). This results in a seven-point scale concerning teacher’s school-level advice. Both the Cito-test score and the school teacher’s advice were measured when the children were 12 years of age and were reported by the parents. Both Cito-test score and the school teacher’s advice are indicators of cognitive performance.

### Potential confounders and covariates

Maternal smoking during pregnancy (any smoking by the mother during pregnancy ≥4 weeks after onset of pregnancy), maternal age at the time of birth in years, sex of the child, birth weight of the child in grams and gestational age in weeks were assessed using questionnaires administrated during pregnancy and the first year of the child’s life.

Parental education level was measured by means of a questionnaire completed by the parents when the children were 1 year old. Both parents were asked to report their educational level separately on a seven-point answering scale (1 = completed primary school; 7 = completed university). Maternal and paternal education was classified into three categories: high, intermediate and low education. Type of child care in the first year (no child care outside home, small-scale child care, day care center attendance) was assessed by a parental report.

Children’s mental health at 12 years of age was assessed using the five-item version of the Mental Health Inventory (MHI-5). The MHI-5 includes questions concerning feeling nervous, calm, happy, depressed and anxious. The scores of five items (ranging from 1 to 5) were added and transformed into a total scoring ranging from 0 to 100. Scores of 60 or less indicate children to be psychological unhealthy, whereas scores of 61 or more indicate good psychological health [18]. Fish consumption (times per week) was assessed by self-report of the parents, and pubertal development was assessed by self-report of the children at the age of 11.

### Statistics

Participant characteristics are presented as means with standard deviations or percentages. The association of fatty acid composition of breast milk with school performance was analyzed with multiple linear regression analyses and multinomial logistic regression. Infant milk feeding fatty acid composition as the independent variable had 3 categories, no n-3 LC-PUFAs (infant formula), “low” in n-3 LC-PUFAs and “high” in n-3 PUFAs, where high was defined as above the median of 0.49 wt% (see online resource 1). The dependent variables were the two indicators for school performance, namely the Cito-test score and school teachers advice. We first analyzed the association between fatty acid composition of breast milk and Cito-test scores. The results are reported as regression coefficients and 95 % CI. The regression coefficient for breast milk can be interpreted as the mean differences (Cito-test score) between low or high fatty acid content and the reference group of no fatty acids (infant formula). In addition, we assessed the linear association between n-3 LC-PUFAs and Cito test by restricting the analyses to breastfed children only and without categorizing n-3 LC-PUFAs. Next we analyzed the association between fatty acid composition of breast milk and school teacher’s advice. The results are reported as OR and 95 % CI. We adjusted all models for variables that were associated with our determinant and outcome, namely parental education level, smoking during pregnancy and child care. The following variables were not associated with the determinant and outcome: gestational age, MHI-5, fish intake of the child, maternal age, birth weight and breastfeeding duration. The analyses were repeated for different types of PUFAs, i.e., DHA, EPA, AA and the ratio of EPA/AA and DHA/AA as independent variables.

We performed analyses stratified for sex to identify different associations within strata.

All statistical analyses were conducted using IBM SPSS (version 20 for windows).

## Results

Children who were never breastfed more often had a smoking mother, lower educated parents, a lower gestational age and attended day care less compared with breastfed children (Table 1). Furthermore, formula-fed children had a lower Cito-test score and got a lower school advice of the teacher compared with breastfed children (Table 2).

**Table 1:** Characteristics of the study population

Variable	Total PIAMA population	Formula	Breastfeeding^c^		*P* value^d^
			“Low” n-3 LC-PUFA	“High” n-3 LC-PUFA	
*n*	3963	277	78	79	
Sex (% girls)	48.2	53.1	48.7	58.2	0.49
Birth weight (g)	3507 ± 546	3499 ± 574	3599 ± 508	3573 ± 493	0.27
Gestational age (weeks)	39.8 ± 1.8	39.6 ± 1.7	40.2 ± 1.2	39.9 ± 1.5	0.003
Maternal age at childbirth (years)	30.4 ± 3.9	30.6 ± 3.7	30.9 ± 3.7	31.5 ± 3.7	0.16
Maternal smoking during pregnancy (%)	17.7	22.2	10.3	5.1	<0.001
Maternal education					<0.001
Low (%)	23.5	34.4	11.5	8.9	
Intermediate (%)	41.6	43.8	43.6	32.9	
Paternal education					<0.001
Low (%)	24.6	37.5	16.9	16.5	
Intermediate (%)	32.7	35.6	23.4	29.1	
Form of child care^a^					0.001
No child care (%)	44.2	37.8	50.0	32.9	
Small child care (%)	31.2	43.3	23.1	30.4	
Large child care (%)	24.6	18.9	26.9	36.7	
*At* ~*11* *years of age*					
Healthy mental health (MHI-5)	93.9 %	94.8 %	94.7 %	92.2 %	0.67
Fish consumption					<0.001
Never (%)	13.2	14.5	17.3	6.7	
Sometimes (%)	47.4	49.4	40.0	54.7	
1–2 times per week (%)	35.6	31.6	40.0	36.0	
3 or more times per week (%)	3.8	4.5	2.7	2.7	
Pubertal development (mean, SD)^b^	1.5 (0.4)	1.6 (0.6)	1.5 (0.5)	1.5 (0.6)	0.44

^a^No child care = no form of child care at all; small child care = regularly cared for by relatives or foster parents and contact with small numbers of children other than their siblings; large child care = child care centers, usually 10 or more children

^b^Puberty developmental scale: mean of 5 items on pubertal development. Answer categories from 1 to 4 (not yet started, barely started, definitely started, seems complete)

^c^“Low” n-3 LC-PUFA was defined as below the median (<0.49 wt%), and “high” n-3 LC-PUFA was defined as above the median (≥0.49 wt%)

^d^ANOVA for continuous variables and Chi-square test for categorical variables

**Table 2:** Descriptive statistics of the school performance of the study population

Variable	Formula	Breastfeeding^b^	
		“Low” n-3 LC-PUFA	“High” n-3 LC-PUFA
*n*	277	78	79
Age (year) at Cito test	11.9 ± 0.3	11.8 ± 0.2	11.9 ± 0.2
Cito-test score (501–550)^a^	537.3 ± 8.4	540.0 ± 7.3	540.8 ± 6.2
School-level advice provided by teachers^a^			
Pre-vocational secondary education (%)	26.0	19.2	10.1
Senior general secondary education (%)	36.1	21.8	34.2
Pre-university education (%)	36.8	56.4	55.7

^a^The Cito test includes questions concerning spelling, math, study skills and world studies. Together the performance scales result in a standardized score between 501 and 550. On average, a pre-vocational secondary education is recommended to children with a Cito-test score between 501 and 536, a senior general secondary education is recommended to children with a score ranging from 537 to 544, and a pre-university education is recommended to children with a score between 544 and 550

^b^“Low” n-3 LC-PUFA was defined as below the median (<0.49 wt%), and “high” n-3 LC-PUFA was defined as above the median (≥0.49 wt%)

Tables 3 and 4 show the association between LC-PUFAs and Cito-test scores as the indicator of school performance. After full adjustment, children who received breast milk with a relatively high amount of DHA had a 1.43 (95 % CI −0.56; 3.42) marginally higher Cito-test score than formula-fed children (Table 3). After full adjustment, girls who received breast milk with a relatively high amount of n-3 LC-PUFAs had a 2.33 (95 % CI −0.32; 4.97) points marginally higher Cito-test score than formula-fed girls (Table 4). This was also the case for the single n-3 LC-PUFA DHA (*β* = 2.96 points higher, 95 % CI 0.24; 5.67). A relatively high amount of all other single n-3 LC-PUFAs and larger DHA/AA and EPA/AA ratios were associated with a higher Cito-test score in girls. There was no association between any of the LC-PUFAs and Cito-test scores in boys (Table 4). Also, there were no associations between a relatively low amount of LC-PUFAs in breast milk and Cito-test scores. Tables 5 and 6 show the association between LC-PUFAs and Cito-test scores within the breastfed children. These tables show similar results to Tables 3 and 4: after full adjustment in girls, for each percentage point of higher total n-3 LC-PUFA content of breast milk, the Cito-test score was 4.55 points (95 % CI 0.43; 8.66) higher. This was also the case for the single n-3 LC-PUFA DHA (*β* = 7.09 points higher, 95 % CI 0.91; 13.27) and the DHA/AA ratio (*β* = 2.33 points higher, 95 % CI 0.02; 4.63). For boys, there was no association between LC-PUFAs and Cito score.

**Table 3:** Difference in test score^a^ between children with low or high LC-PUFAs compared to formula-fed children (no LC-PUFAs)

	Unadjusted	Adjusted^b^
*Total n*-*3 LC*-*PUFA*		
No breastfeeding	0 (ref)	0 (ref)
Breastfeeding n-3 LC-PUFA content less than median^c^	2.64 (0.65, 4.62)*	0.75 (−1.25, 2.75)
Breastfeeding n-3 LC-PUFA content at/more than median	3.52 (1.55, 5.50)*	1.02 (−1.00, 3.04)
*DHA*		
No breastfeeding	0 (ref)	0 (ref)
Breastfeeding DHA content less than median^c^	2.52 (0.52, 4.51)*	0.32 (−1.70, 2.34)
Breastfeeding DHA content at or more than median	3.63 (1.66, 5.59)*	1.43 (−0.56, 3.42)
*EPA*		
No breastfeeding	0 (ref)	0 (ref)
Breastfeeding EPA content less than median^c^	2.95 (0.98, 4.93)*	1.05 (−0.94, 3.03)
Breastfeeding EPA content at or more than median	3.21 (1.23, 5.20)*	0.71 (−1.32, 2.74)
*AA*		
No breastfeeding	0 (ref)	0 (ref)
Breastfeeding AA content less than median^c^	2.69 (0.71, 4.66)*	0.50 (−1.50, 2.50)
Breastfeeding AA content at or more than median	3.48 (1.50, 5.47)*	1.27 (−0.73, 3.26)
*DHA/AA ratio*		
No breastfeeding	0 (ref)	0 (ref)
Breastfeeding DHA/AA ratio less than median^c^	3.46 (1.47, 5.45)*	0.40 (−1.64, 2.43)
Breastfeeding DHA/AA ratio at or more than median	2.66 (0.67, 4.65)*	1.25 (−0.75, 3.26)
*EPA/AA ratio*		
No breastfeeding	0 (ref)	0 (ref)
Breastfeeding EPA/AA ratio less than median^c^	3.56 (1.57, 5.55)*	0.73 (−1.26, 2.72)
Breastfeeding EPA/AA ratio at or more than median	2.56 (0.57, 4.55)*	0.95 (−1.09, 2.99)

* Significant *P* < 0.05

^a^Range 501–550

^b^Adjusted for parental educational level, smoking during pregnancy and child care

^c^Median was 0.49 wt% for n-3 LC-PUFA, 0.17 wt% for DHA, 0.04 wt% for EPA, 0.37 wt% for AA, 0.44 for DHA/AA ratio and 0.07 for EPA/DHA ratio

**Table 4:** Difference in test score^a^ between children with low or high LC-PUFAs compared to formula-fed children (no LC-PUFAs)

	Girls		Boys	
	Unadjusted	Adjusted^b^	Unadjusted	Adjusted^b^
*Total n*-*3 LC*-*PUFA*				
No breastfeeding	0 (ref)	0 (ref)	0 (ref)	0 (ref)
Breastfeeding n-3 LC-PUFA content less than median^c^	2.37 (−0.35, 5.09)	0.92 (−1.91, 3.74)	2.88 (−0.05, 5.82)	0.54 (−2.39, 3.48)
Breastfeeding n-3 LC-PUFA content at/more than median	4.50 (1.98, 7.03)*	2.33 (−0.32, 4.97)	2.16 (−1.00, 5.33)	−0.83 (−4.04, 2.38)
*DHA*				
No breastfeeding	0 (ref)	0 (ref)	0 (ref)	0 (ref)
Breastfeeding DHA content less than median^c^	2.43 (−0.18, 5.05)	0.41 (−2.32, 3.14)	2.62 (−0.47, 5.71)	0.28 (−2.82, 3.38)
Breastfeeding DHA content at or more than median	4.65 (2.03, 7.26)*	2.96 (0.24, 5.69)*	2.50 (−0.50, 5.49)	−0.37 (−3.39, 2.65)
*EPA*				
No breastfeeding	0 (ref)	0 (ref)	0 (ref)	0 (ref)
Breastfeeding EPA content less than median^c^	2.62 (−0.05, 5.29)	1.02 (−1.75, 3.79)	3.30 (0.34, 6.26)*	1.02 (−1.89, 3.94)
Breastfeeding EPA content at or more than median	4.38 (1.81, 6.95)*	2.30 (−0.39, 4.99)	1.71 (−1.42, 4.83)	−1.43 (−4.61, 1.75)
*AA*				
No breastfeeding	0 (ref)	0 (ref)	0 (ref)	0 (ref)
Breastfeeding AA content less than median^c^	2.74 (0.10, 5.39)*	0.82 (−1.93, 3.57)	2.63 (−0.37, 5.62)	0.12 (−2.90, 3.14)
Breastfeeding AA content at or more than median	4.30 (1.70, 6.90)*	2.48 (−0.19, 5.15)	2.48 (−0.61, 5.57)	−0.25 (−3.35, 2.85)
*DHA/AA ratio*				
No breastfeeding	0 (ref)	0 (ref)	0 (ref)	0 (ref)
Breastfeeding DHA/AA ratio less than median^c^	2.39 (−0.28, 5.06)	0.67 (−2.11, 3.46)	2.14 (−0.96, 5.23)	0.09 (−3.00, 3.18)
Breastfeeding DHA/AA ratio at or more than median	4.53 (1.94, 7.13)*	2.46 (−0.25, 5.16)	2.95 (−0.05, 5.94)	−0.20 (−3.26, 2.86)
*EPA/AA ratio*				
No breastfeeding	0 (ref)	0 (ref)	0 (ref)	0 (ref)
Breastfeeding EPA/AA ratio less than median^c^	2.23 (−0.47, 4.92)	0.95 (−1.82, 3.71)	2.18 (−0.95, 5.31)	0.55 (−2.39, 3.49)
Breastfeeding EPA/AA ratio at or more than median	4.63 (2.06, 7.20)*	2.24 (−0.49, 4.98)	2.89 (−0.08, 5.85)	−0.81 (−3.99, 2.37)

* Significant *P* < 0.05

^a^Range 501–550

^b^Adjusted for parental educational level, smoking during pregnancy and child care

^c^Median was 0.49 wt% for n-3 LC-PUFA, 0.17 wt% for DHA, 0.04 wt% for EPA, 0.37 wt% for AA, 0.44 for DHA/AA ratio and 0.07 for EPA/DHA ratio

**Table 5:** Association between LC-PUFAs in breast milk and test score^a^

	Unadjusted	Adjusted^b^
Total n-3 LC-PUFA	3.48 (0.02; 6.94)*	1.77 (−1.61; 5.16)
DHA	5.73 (0.31; 11.14)*	3.73 (−1.54; 9.00)
EPA	12.83 (−7.36; 33.01)	5.00 (−14.32; 24.32)
AA	7.79 (−4.60; 20.18)	6.27 (−5.47; 18.01)
DHA/AA ratio	2.00 (0.00; 4.00)*	1.27 (−0.67; 3.21)
EPA/AA ratio	4.43 (−2.99; 11.85)	1.70 (−5.37; 8.76)

* Significant *P* < 0.05

^a^Range 501–550

^b^Adjusted for parental educational level, smoking during pregnancy and child care

**Table 6:** Association between LC-PUFAs in breast milk and test score^a^, for boys and girls

	Girls		Boys	
	Unadjusted	Adjusted^b^	Unadjusted	Adjusted^b^
Total n-3 LC-PUFA	4.45 (0.72; 8.19)*	4.55 (0.43; 8.66)*	0.16 (−7.38; 7.70)	−0.10 (−8.94; 2.89)
DHA	6.84 (1.14; 12.55)*	7.09 (0.91; 13.27)*	0.95 (−11.79; 13.67)	−3.47 (−13.75; 6.82)
EPA	24.73 (1.99; 47.46)*	24.41 (−0.04; 48.85)	−12.72 (−51.13; 25.68)	−22.95 (−53.06; 7.16)
AA	9.68 (−5.36; 24.71)	9.64 (−5.82; 25.09)	0.04 (−17.5; 25.50)	−2.88 (−20.72; 14.97)
DHA/AA ratio	2.37 (0.21; 4.53)*	2.33 (0.02; 4.63)*	0.73 (−3.63; 5.09)	−0.49 (−4.03; 3.06)
EPA/AA ratio	8.42 (0.03; 16.81)*	7.98 (−0.91; 16.87)	−4.04 (−18.13; 10.05)	−6.41 (−17.51; 4.68)

* Significant *P* < 0.05

^a^Range 501–550

^b^Adjusted for parental educational level, smoking during pregnancy and child care

The association between LC-PUFAs and teacher’s school advice regarding the suitable school level for a child as the indicator of school performance showed a similar pattern (online resource 2). A higher proportion of girls who received breast milk with a relatively high amount of total n-3 LC-PUFAs, DHA, EPA, DHA/AA ratio and EPA/AA ratio get a higher school-level advice compared to formula-fed girls (online resource 3). However, after full adjustment, the associations attenuated to nonsignificant. There were no associations between LC-PUFAs and teacher’s school advice for boys (online resource 4) and in children who received a relatively low amount of n-3 LC-PUFAs in breast milk.

## Discussion

### Summary of main findings

This study shows that girls receiving breast milk with a relatively high content of DHA had a significantly better school performance at age 12 years after adjustment for confounding factors than formula-fed girls. A similar tendency was observed for the breast milk content of total n-3 LC-PUFA, EPA, AA and the ratio of DHA/AA and EPA/DHA, but these associations were not statistically significant after adjustment for potential confounders. There was no association between LC-PUFA content of breast milk and school performance in boys.

### Comparison with literature and underlying mechanism

It has been observed before that breast feeding is associated with better cognitive performance but the mechanism is unclear. However, n-3 and n-6 LC-PUFAs are important components of the cell membranes and, therefore, essential in the formation of new tissue, including neurons and glial cells [19, 20]. In addition, PUFA may have a neuroprotective role, making nervous tissue less susceptible to damage [21]. Moreover, EPA, DHA and AA can act as secondary messengers and also induce release of acetylcholine and noradrenaline. These neurotransmitters are involved in memory and learning and may play a role during development [22, 23]. Taken together, all these factors, brain components, neuroprotection and neurotransmission, may play a role in the association between LC-PUFA content of breast milk and cognitive performance at 12 years of age. So far only the association between cognitive performance and LC-PUFA content of breast milk in very young children has been investigated [10, 11]. Gustafsson et al. [11] showed in a very small sample (*n* = 28) that high colostrum LC-PUFAs levels were associated with better cognitive performance at the age of 6.5 years. However, breast milk samples after 1 and 3 months did not disclose an association between LC-PUFA content and cognitive performance in this study. In addition, a recent study reported that higher levels of n-3 LC-PUFAs and higher ratios between n-3 and n-6 LC-PUFAs in breast milk were positively associated with cognitive performance in children at 14 months of age [10]. We have extended these findings by showing that a high content of LC-PUFA in breast milk is associated with better school performance among girls at 12 years of age.

Although the previous mentioned studies did not assess sex differences in the association between breast milk fatty acids and cognition, earlier studies have assessed whether the association between supplementation of LC-PUFAs in infant formulas and cognition differs according to the child’s sex. Our results are in line with these results reporting a beneficial effect for girls [24, 25]. Although it has been shown that fatty acid metabolism is different between men and women [26], there is yet no satisfactory explanation for these sex differences in children. We can only speculate about possible mechanisms. First, the metabolic difference between men and women may also be present in children, although metabolic differences seem to be driven by sex hormones [26] which may not apply to infants. Second, environmental influences have been shown to increase at later age [27]. These influences may be larger for boys than girls. Third, there is an age-specific development of cognitive function [28], and therefore, at the age of 12, our outcome measurement may have been more sensitive for girls than for boys.

### Methodological considerations

Strengths of this study include the prospective study design and the availability of LC-PUFA content of breast milk. However, some limitations have to be considered. We performed multiple comparisons with related exposure variables, which increase the chance that a statistically significant estimate is found. No adequate method for adjustment for multiple comparisons exists. Therefore, we think it is important to interpret our findings based on effect size and consistency.

Because we obtained only one sample of breast milk, it is not known whether the fatty acid composition in the breast milk sample reflects the fatty acid composition of the breast milk of the entire lactation period. However, breast milk fatty acid composition reflects long-term food intake by the mother [29] and predicts infant DHA status [30]. Moreover, we found no correlation between breast milk fatty acids and the time between delivery and when the samples were obtained. If misclassification occurred, this misclassification is, however, unlikely to be differentially associated with later school performance; that is, it is unlikely that all of the mothers with a lower than their usual content of n-3 LC-PUFAs in their milk on the day they collected the sample will have children with a higher school performance at age 12 years.

We collected data on fatty acid composition of breast milk in a subsample of the PIAMA study. The mothers who were willing to donate a milk sample had higher education and were less often smokers than those who did not breastfeed. However, we consider it to be unlikely that the association between breast milk fatty acid content and school performance at age 12 is different in the group that we studied than in the total study population. Also, because of the subsample we used, we were only able to compare two groups of breastfeeding (by median split). As indicated by the width of the confidence intervals, our sample size was sufficient to perform analyses stratified by sex, and adjustment for the main confounders, but our sample size was not sufficient to create more than 2 groups.

Adjustment for the confounders resulted in a large attenuation of the estimates, especially in the analyses where infant formula was used as the reference group. Estimates between crude and adjusted analysis within the breastfed group were more similar, as confounding by lifestyle and sociodemographic factors determining choice of infant milk feeding was not an issue in this analysis. Thus, residual confounding by lifestyle factors can never be ruled out, especially in the analyses where formula-fed children were used as the reference. On the other hand, in unadjusted analyses, an association between n-3 LC-PUFAs and cognition was seen in boys and girls, while after adjustment, only associations in girls were seen. Residual confounding should then include sex-specific confounders. Sex-specific confounding is unlikely, and therefore, residual confounding might not completely explain the reported association.

## Implications

We showed, at least in girls, that infant feeding is important for long-term cognitive performance at least till the age of 12. Other studies found that better cognition in childhood predicts better cognition in adulthood. As cognition is also associated with socioeconomic status and because of the strong associations between socioeconomic status and health, perhaps improving cognition in childhood may eventually benefit population health.

Therefore, our findings call for novel, well-powered RCTs that verify the effect of LC-PUFA supplementation or intake on the breast milk content in relation to child cognitive performance.

## Conclusion

Although a large part of the association between infant milk feeding and cognition seems to be explained by sociodemographic and lifestyle-related factors, a relative high content of n-3 PUFAs, especially DHA, in breast milk is associated with better school performance in 12-year-old girls but not in boys.

## Electronic supplementary material

Below is the link to the electronic supplementary material.

Supplementary material 1 (DOC 123 kb)

## References

[1] Eidelman AI, Schanler RJ (2012). Breastfeeding and the use of human milk. Pediatrics.

[2] Daniels JL, Longnecker MP, Rowland AS, Golding J (2004). Fish intake during pregnancy and early cognitive development of offspring. Epidemiology.

[3] Oken E, Radesky JS, Wright RO, Bellinger DC, Amarasiriwardena CJ, Kleinman KP, Hu H, Gillman MW (2008). Maternal fish intake during pregnancy, blood mercury levels, and child cognition at age 3 years in a US cohort. Am J Epidemiol.

[4] Hibbeln JR, Davis JM, Steer C, Emmett P, Rogers I, Williams C, Golding J (2007). Maternal seafood consumption in pregnancy and neurodevelopmental outcomes in childhood (ALSPAC study): an observational cohort study. Lancet.

[5] Mendez MA, Torrent M, Julvez J, Ribas-Fito N, Kogevinas M, Sunyer J (2009). Maternal fish and other seafood intakes during pregnancy and child neurodevelopment at age 4 years. Public Health Nutr.

[6] Gould JF, Smithers LG, Makrides M (2013). The effect of maternal omega-3 (n-3) LCPUFA supplementation during pregnancy on early childhood cognitive and visual development: a systematic review and meta-analysis of randomized controlled trials. Am J Clin Nutr.

[7] Simmer K, Patole SK, Rao SC (2011) Long-chain polyunsaturated fatty acid supplementation in infants born at term. Cochrane Database Syst Rev (12):CD000376. doi:10.1002/14651858.CD000376.pub3 10.1002/14651858.CD000376.pub2 18253974

[8] Schulzke SM, Patole SK, Simmer K (2011) Long-chain polyunsaturated fatty acid supplementation in preterm infants. Cochrane Database Syst Rev (2):CD000375. doi:10.1002/14651858.CD000375.pub4 10.1002/14651858.CD000375.pub3 18253973

[9] Meldrum SJ, Smith MA, Prescott SL, Hird K, Simmer K (2011). Achieving definitive results in long-chain polyunsaturated fatty acid supplementation trials of term infants: factors for consideration. Nutr Rev.

[10] Guxens M, Mendez MA, Molto-Puigmarti C, Julvez J, Garcia-Esteban R, Forns J, Ferrer M, Vrijheid M, Lopez-Sabater MC, Sunyer J (2011). Breastfeeding, long-chain polyunsaturated fatty acids in colostrum, and infant mental development. Pediatrics.

[11] Gustafsson PA, Duchen K, Birberg U, Karlsson T (2004). Breastfeeding, very long polyunsaturated fatty acids (PUFA) and IQ at 6 1/2 years of age. Acta Paediatr.

[12] Decsi T, Kennedy K (2011). Sex-specific differences in essential fatty acid metabolism. Am J Clin Nutr.

[13] Lim M, Mathus-Vliegen EMH (1996). De voedingsmiddelengids (the nutrition guide).

[14] Wijga AH, Kerkhof M, Gehring U, de Jongste JC, Postma DS, Aalberse RC, Wolse AP, Koppelman GH, van RL, Oldenwening M, Brunekreef B, Smit HA (2014). Cohort profile: the prevention and incidence of asthma and mite allergy (PIAMA) birth cohort. Int J Epidemiol.

[15] Foreman-van Drongelen MM, Houwelingen AC, Kester AD, de Jong AE, Blanco CE, Hasaart TH, Hornstra G (1995). Long-chain polyene status of preterm infants with regard to the fatty acid composition of their diet: comparison between absolute and relative fatty acid levels in plasma and erythrocyte phospholipids. Br J Nutr.

[16] Wijga A, Houwelingen AC, Smit HA, Kerkhof M, Vos AP, Neijens HJ, Brunekreef B (2003). Fatty acids in breast milk of allergic and non-allergic mothers: the PIAMA birth cohort study. Pediatr Allergy Immunol.

[17] Cito (2013) betekenis van de standaardscore op de citotoets. http://www.cito.nl/

[18] Perenboom R, Oudshoorn K, Herten L, Hoeymans N, Bijl RV (2000) Levensverwachting in goede geestelijke gezondheid: bepaling afkappunten in wegingsfactoren voor de MHI-5 en GHQ-12. Leiden, TNO

[19] Hornstra G (2000). Essential fatty acids in mothers and their neonates. Am J Clin Nutr.

[20] Jamieson EC, Abbasi KA, Cockburn F, Farquharson J, Logan RW, Patrick WA (1994). Effect of diet on term infant cerebral cortex fatty acid composition. World Rev Nutr Diet.

[21] Lauritzen I, Blondeau N, Heurteaux C, Widmann C, Romey G, Lazdunski M (2000). Polyunsaturated fatty acids are potent neuroprotectors. EMBO J.

[22] Das UN (2003). Long-chain polyunsaturated fatty acids in the growth and development of the brain and memory. Nutrition.

[23] Farkas E, de Wilde MC, Kiliaan AJ, Meijer J, Keijser JN, Luiten PG (2002). Dietary long chain PUFAs differentially affect hippocampal muscarinic 1 and serotonergic 1A receptors in experimental cerebral hypoperfusion. Brain Res.

[24] Makrides M, Gibson RA, McPhee AJ, Collins CT, Davis PG, Doyle LW, Simmer K, Colditz PB, Morris S, Smithers LG, Willson K, Ryan P (2009). Neurodevelopmental outcomes of preterm infants fed high-dose docosahexaenoic acid: a randomized controlled trial. JAMA.

[25] Isaacs EB, Ross S, Kennedy K, Weaver LT, Lucas A, Fewtrell MS (2011). 10-year cognition in preterms after random assignment to fatty acid supplementation in infancy. Pediatrics.

[26] Burdge GC, Calder PC (2005). Conversion of alpha-linolenic acid to longer-chain polyunsaturated fatty acids in human adults. Reprod Nutr Dev.

[27] Schneider LA, Burns NR, Giles LC, Higgins RD, Nettelbeck TJ, Ridding MC, Pitcher JB (2014). Cognitive abilities in preterm and term-born adolescents. J Pediatr.

[28] Asato MR, Terwilliger R, Woo J, Luna B (2010). White matter development in adolescence: a DTI study. Cereb Cortex.

[29] Jensen RG (1999). Lipids in human milk. Lipids.

[30] Dunstan JA, Mitoulas LR, Dixon G, Doherty DA, Hartmann PE, Simmer K, Prescott SL (2007). The effects of fish oil supplementation in pregnancy on breast milk fatty acid composition over the course of lactation: a randomized controlled trial. Pediatr Res.

