# Supplementary material for: Fatty acid composition in breastfeeding and school performance in children aged 12 years
Source: Eur J Nutr. 2015 Sep 7;55(7):2199–207. doi: 10.1007/s00394-015-1030-y (PMC5035317; doi:10.1007/s00394-015-1030-y)
Supplement: Supplementary file 1 — Supplementary material 1 (DOC 123 kb) [file 394_2015_1030_MOESM1_ESM.doc]

**ONLINE RESOURCE**

**Fatty acid composition in breastfeeding and school performance in children aged 12 years**

Geertje W. Dalmeijer^1^, Alet H. Wijga^2^, Ulrike Gehring^3^, Carry M. Renders^4^, Gerard H. Koppelman^5^, Henriette A. Smit^1^, Lenie van Rossem^1^

**Affiliations:**

^1^Julius Center for Health Sciences and Primary Care, University Medical Center Utrecht, the Netherlands

^2^National Institute for Public Health and the Environment (RIVM), Bilthoven, Netherlands

^3^ Institute for Risk Assessment Sciences, Utrecht University, Utrecht, The Netherlands

^4^Department of Health Sciences Faculty of Earth and Life Sciences, and EMGO Institute for Health and Care Research, VU University Amsterdam, the Netherlands

^5^University of Groningen, University Medical Center Groningen, Beatrix Children’s Hospital, Dpt of Pediatric Pulmonology and Pediatric Allergology, and Groningen Research Institute for Asthma and COPD

**Address correspondence to:**

L. van Rossem, PhD

Julius Center for Health Sciences and Primary Care, University Medical Center Utrecht

STRT 6.131, PO Box 85500, 3508GA Utrecht, The Netherlands

🕿0031 88 75 68202, 🖂 [L.vanRossem@umcutrecht.nl](mailto:L.vanRossem@umcutrecht.nl)

Online Resource 1: Fatty acid composition (percentage of total fatty acids) of breast milk at age 12 years

|  | PUFA levels | Low^1^ PUFA levels | High^2^ PUFA levels |
| --- | --- | --- | --- |
|  | Mean wt% (SD) | Mean wt% (SD) | Mean wt% (SD) |
| **N-3 PUFA’s** |  |  |  |
| Total n-3 PUFA’s | 0.54 (0.31) | 0.38 (0.09) | 0.70 (0.36) |
| DHA | 0.22 (0.19) | 0.12 (0.03) | 0.31 (0.24) |
| EPA | 0.05 (0.05) | 0.02(0.01) | 0.08 (0.06) |
| **n-6 PUFA** |  |  |  |
| AA | 0.37 (0.09) | 0.30 (0.06) | 0.44 (0.05) |
| **n-3/n-6 PUFA’s ratio** |  |  |  |
| DHA/AA ratio | 0.59 (0.53) | 0.35 (0.09) | 0.83 (0.67) |
| EPA/AA ratio | 0.07 (0.02) | 0.05 (0.01) | 0.09 (0.02) |

AA indicates arachidonic acid; DHA docosahexaenoic; EPA, eicosapentaenoic; LC-PUFA, long-chain poly unsaturated fatty acids; wt, weight

^1^ Breast milk PUFA levels less than median

^2^ Breast milk PUFA levels at or more than median

**Online Resource 2: Association between n-3 LC PUFA’s in breast milk and teacher’s school level advice**

|  | Unadjusted Odds Ratios | | | Adjusted^a^ Odds Ratios | | |
| --- | --- | --- | --- | --- | --- | --- |
|  | Prevocational secondary education | Senior general secondary education | Pre-university education | Prevocational secondary education | Senior general secondary education | Pre-university education |
| **n-3 LC-PUFA** |  |  |  |  |  |  |
| No breastfeeding | 1 (ref) | 1 (ref) | 1 (ref) | 1 (ref) | 1 (ref) | 1 (ref) |
| Breastfeeding n-3 LC-PUFA content less than median^b^ | 1 (ref) | 0.82 (0.38, 1.74) | 2.07 (1.07, 4.00) | 1 (ref) | 0.71 (0.31, 1.67) | 1.15 (0.53, 2.50) |
| Breastfeeding n-3 LC-PUFA content at or more than median | 1 (ref) | 2.43 (1.04, 5.66) | 3.88 (1.73, 8.74) | 1 (ref) | 1.75 (0.70, 4.34) | 1.85 (0.75, 4.53) |
| **DHA** |  |  |  |  |  |  |
| No breastfeeding | 1 (ref) | 1 (ref) | 1 (ref) | 1 (ref) | 1 (ref) | 1 (ref) |
| Breastfeeding DHA content less than median^b^ | 1 (ref) | 1.18 (0.57, 2.46) | 1.92 (0.97, 3.79) | 1 (ref) | 0.89 (0.39, 2.07) | 0.86 (0.38, 1.95) |
| Breastfeeding DHA content at or more than median | 1 (ref) | 1.68 (0.73, 3.88) | 3.92 (1.81, 8.48) | 1 (ref) | 1.38 (0.56, 3.39) | 2.26 (0.96, 5.32) |
| **EPA** |  |  |  |  |  |  |
| No breastfeeding | 1 (ref) | 1 (ref) | 1 (ref) | 1 (ref) | 1 (ref) | 1 (ref) |
| Breastfeeding EPA content less than median^b^ | 1 (ref) | 0.59 (0.28, 1.28) | 1.95 (1.04, 3.67) | 1 (ref) | 0.48 (0.20, 1.12) | 1.03 (0.49, 2.18) |
| Breastfeeding EPA content at or more than median | 1 (ref) | 3.60 (1.42, 9.10) | 4.82 (1.95, 11.96) | 1 (ref) | 2.78 (1.04, 7.45) | 2.40 (0.89, 6.48) |
| **AA** |  |  |  |  |  |  |
| No breastfeeding | 1 (ref) | 1 (ref) | 1 (ref) | 1 (ref) | 1 (ref) | 1 (ref) |
| Breastfeeding AA content less than median^b^ | 1 (ref) | 2.52 (1.09, 5.85) | 3.62 (1.60, 8.18) | 1 (ref) | 2.17 (0.86, 5.49) | 1.95 (0.78, 4.88) |
| Breastfeeding AA content at or more than median | 1 (ref) | 0.77 (0.36, 1.65) | 2.21 (1.15, 4.26) | 1 (ref) | 0.60 (0.26, 1.38) | 1.13 (0.53, 2.41) |
| **DHA/AA ratio** |  |  |  |  |  |  |
| No breastfeeding | 1 (ref) | 1 (ref) | 1 (ref) | 1 (ref) | 1 (ref) | 1 (ref) |
| Breastfeeding DHA/AA ratio less than median^b^ | 1 (ref) | 0.96 (0.46, 2.00) | 1.93 (0.99, 3.75) | 1 (ref) | 0.75 (0.33, 1.74) | 0.88 (0.40, 1.94) |
| Breastfeeding DHA/AA ratio at or more than median | 1 (ref) | 2.16 (0.92, 5.08) | 4.06 (1.81, 9.12) | 1 (ref) | 1.71 (0.68, 4.29) | 2.28 (0.93, 5.59) |
| **EPA/AA ratio** |  |  |  |  |  |  |
| No breastfeeding | 1 (ref) | 1 (ref) | 1 (ref) | 1 (ref) | 1 (ref) | 1 (ref) |
| Breastfeeding EPA/AA ratio less than median^b^ | 1 (ref) | 0.49 (0.23, 1.06) | 1.67 (0.90, 3.09) | 1 (ref) | 0.41 (0.18, 0.97) | 0.92 (0.44, 1.91) |
| Breastfeeding EPA/AA ratio at or more than median | 1 (ref) | 5.58 (1.89, 16.51)* | 7.41 (2.55, 21.59)* | 1 (ref) | 4.16 (1.33, 12.96)* | 3.49 (1.12, 10.92)* |

^a^Adjusted for parental educational level, smoking during pregnancy, and child care.

^b^ Median was 0.49 wt% for n-3 LC-PUFA, 0.17 wt% for DHA, 0.04 wt% for EPA, 0.37 wt% for AA, 0.44 for DHA/AA ratio and 0.07 for EPA/DHA ratio

**Online Resource 3: Association between n-3 LC PUFA’s in breast milk and teacher’s school level advice for girls**

|  | Unadjusted Odds Ratios | | | Adjusted^a^ Odds Ratios | | |
| --- | --- | --- | --- | --- | --- | --- |
|  | Prevocational secondary education | Senior general secondary education | Pre-university education | Prevocational secondary education | Senior general secondary education | Pre-university education |
| **n-3 LC-PUFA** |  |  |  |  |  |  |
| No breastfeeding | 1 (ref) | 1 (ref) | 1 (ref) | 1 (ref) | 1 (ref) | 1 (ref) |
| Breastfeeding n-3 LC-PUFA content less than median^b^ | 1 (ref) | 1.02 (0.36, 2.88) | 1.70 (0.65, 4.45) | 1 (ref) | 1.01 (0.30, 3.35) | 0.90 (0.28., 2.85) |
| Breastfeeding n-3 LC-PUFA content at or more than median | 1 (ref) | 2.43 (0.75, 7.93) | 4.22 (1.36, 13.08) | 1 (ref) | 1.85 (0.52, 6.65) | 2.00 (0.57, 6.95) |
| **DHA** |  |  |  |  |  |  |
| No breastfeeding | 1 (ref) | 1 (ref) | 1 (ref) | 1 (ref) | 1 (ref) | 1 (ref) |
| Breastfeeding DHA content less than median^b^ | 1 (ref) | 1.48 (0.55, 3.97) | 1.61 (0.61, 4.24) | 1 (ref) | 1.27 (0.38, 4.15) | 0.64 (0.19, 2.09) |
| Breastfeeding DHA content at or more than median | 1 (ref) | 1.62 (0.47, 5.57) | 4.38 (1.41, 13.54) | 1 (ref) | 1.42 (0.38, 5.36) | 2.73 (0.78, 9.60) |
| **EPA** |  |  |  |  |  |  |
| No breastfeeding | 1 (ref) | 1 (ref) | 1 (ref) | 1 (ref) | 1 (ref) | 1 (ref) |
| Breastfeeding EPA content less than median^b^ | 1 (ref) | 0.65 (0.23, 1.79) | 1.46 (0.60, 3.54) | 1 (ref) | 0.56 (0.17, 1.85) | 0.6 (0.23, 2.02) |
| Breastfeeding EPA content at or more than median | 1 (ref) | 5.51 (1.20, 25.33) | 7.81 (1.74, 35.05) | 1 (ref) | 4.42 (0.90, 21.82) | 4.04 (0.82, 19.87) |
| **AA** |  |  |  |  |  |  |
| No breastfeeding | 1 (ref) | 1 (ref) | 1 (ref) | 1 (ref) | 1 (ref) | 1 (ref) |
| Breastfeeding AA content less than median^b^ | 1 (ref) | 2.59 (0.80, 8.40) | 3.13 (0.99, 9.91) | 1 (ref) | 2.44 (0.66, 9.10) | 1.54 (0.42, 5.63) |
| Breastfeeding AA content at or more than median | 1 (ref) | 0.93 (0.32, 2.66) | 2.32 (0.91, 5.92) | 1 (ref) | 0.79 (0.25, 2.56) | 1.24 (0.42, 3.65) |
| **DHA/AA ratio** |  |  |  |  |  |  |
| No breastfeeding | 1 (ref) | 1 (ref) | 1 (ref) | 1 (ref) | 1 (ref) | 1 (ref) |
| Breastfeeding DHA/AA ratio less than median^b^ | 1 (ref) | 1.39 (0.52, 3.75) | 1.52 (0.57, 4.03) | 1 (ref) | 1.60 (0.48, 5.36) | 0.80 (0.24, 2.69) |
| Breastfeeding DHA/AA ratio at or more than median | 1 (ref) | 1.78 (0.53, 6.04) | 4.38 (1.41, 13.54) | 1 (ref) | 1.65 (0.43, 6.32) | 2.34 (0.66, 8.32) |
| **EPA/AA ratio** |  |  |  |  |  |  |
| No breastfeeding | 1 (ref) | 1 (ref) | 1 (ref) | 1 (ref) | 1 (ref) | 1 (ref) |
| Breastfeeding EPA/AA ratio less than median^b^ | 1 (ref) | 0.65 (0.23, 1.79) | 1.39 (0.57, 3.39) | 1 (ref) | 0.61 (0.19, 1.97) | 0.76 (0.26, 2.22) |
| Breastfeeding EPA/AA ratio at or more than median | 1 (ref) | 5.51 (1.20, 25.33)* | 7.81 (1.74, 35.05)* | 1 (ref) | 4.23 (0.85, 21.07) | 3.32 (0.67, 16.45) |

^a^Adjusted for parental educational level, smoking during pregnancy, and child care

^b^ Median was 0.49 wt% for n-3 LC-PUFA, 0.17 wt% for DHA, 0.04 wt% for EPA, 0.37 wt% for AA, 0.44 for DHA/AA ratio and 0.07 for EPA/DHA ratio

Online Resource 4: Association between n-3 LC PUFA’s in breast milk and teacher’s school level advice for boys

|  | Unadjusted Odds Ratios | | | Adjusted^a^ Odds Ratios | | |
| --- | --- | --- | --- | --- | --- | --- |
|  | Prevocational secondary education | Senior general secondary education | Pre-university education | Prevocational secondary education | Senior general secondary education | Pre-university education |
| **n-3 LC-PUFA** |  |  |  |  |  |  |
| No breastfeeding | 1 (ref) | 1 (ref) | 1 (ref) | 1 (ref) | 1 (ref) | 1 (ref) |
| Breastfeeding n-3 LC-PUFA content less than median^b^ | 1 (ref) | 0.60 (0.19, 1.89) | 2.51 (1.02, 6.22) | 1 (ref) | 0.37 (0.11, 1.28) | 1.17 (0.40, 3.37) |
| Breastfeeding n-3 LC-PUFA content at or more than median | 1 (ref) | 2.41 (0.72, 8.11) | 3.42 (1.06, 11.04) | 1 (ref) | 1.40 (0.38, 5.16) | 1.38 (0.37, 5.16) |
| **DHA** |  |  |  |  |  |  |
| No breastfeeding | 1 (ref) | 1 (ref) | 1 (ref) | 1 (ref) | 1 (ref) | 1 (ref) |
| Breastfeeding DHA content less than median^b^ | 1 (ref) | 0.80 (0.26, 2.56) | 2.30 (0.88, 6.02) | 1 (ref) | 0.47 (0.14, 1.62) | 1.02 (0.33, 3.16) |
| Breastfeeding DHA content at or more than median | 1 (ref) | 1.77 (0.57, 5.55) | 3.54 (1.22, 10.25) | 1 (ref) | 1.07 (0.31, 3.64) | 1.53 (0.46, 5.05) |
| **EPA** |  |  |  |  |  |  |
| No breastfeeding | 1 (ref) | 1 (ref) | 1 (ref) | 1 (ref) | 1 (ref) | 1 (ref) |
| Breastfeeding EPA content less than median^b^ | 1 (ref) | 0.50 (0.15, 1.67) | 2.61 (1.06, 6.45) | 1 (ref) | 0.32 (0.09, 1.14) | 1.31 (0.46, 3.72) |
| Breastfeeding EPA content at or more than median | 1 (ref) | 2.61 (0.79, 8.69) | 3.22 (0.99, 10.45) | 1 (ref) | 1.50 (0.41, 5.46) | 1.15 (0.30, 4.37) |
| **AA** |  |  |  |  |  |  |
| No breastfeeding | 1 (ref) | 1 (ref) | 1 (ref) | 1 (ref) | 1 (ref) | 1 (ref) |
| Breastfeeding AA content less than median^b^ | 1 (ref) | 2.41 (0.72, 8.11) | 4.22 (1.33, 13.38) | 1 (ref) | 1.59 (0.43, 5.79) | 2.11 (0.58, 7.63) |
| Breastfeeding AA content at or more than median | 1 (ref) | 0.60 (0.19, 1.89) | 2.11 (0.84, 5.31) | 1 (ref) | 0.32 (0.09, 1.12) | 0.81 (0.27, 2.39) |
| **DHA/AA ratio** |  |  |  |  |  |  |
| No breastfeeding | 1 (ref) | 1 (ref) | 1 (ref) | 1 (ref) | 1 (ref) | 1 (ref) |
| Breastfeeding DHA/AA ratio less than median^b^ | 1 (ref) | 0.50 (0.15, 1.67) | 2.41 (0.97, 5.99) | 1 (ref) | 0.27 (0.07, 1.03) | 0.88 (0.28, 2.74) |
| Breastfeeding DHA/AA ratio at or more than median | 1 (ref) | 2.61 (0.79, 8.69) | 3.62 (1.13, 11.62) | 1 (ref) | 1.85 (0.51, 6.71) | 2.07 (0.57, 7.55) |
| **EPA/AA ratio** |  |  |  |  |  |  |
| No breastfeeding | 1 (ref) | 1 (ref) | 1 (ref) | 1 (ref) | 1 (ref) | 1 (ref) |
| Breastfeeding EPA/AA ratio less than median^b^ | 1 (ref) | 0.32 (0.09, 1.11) | 2.01 (0.86, 4.71) | 1 (ref) | 0.20 (0.05, 0.74) | 0.94 (0.34, 2.59) |
| Breastfeeding EPA/AA ratio at or more than median | 1 (ref) | 5.63 (1.20, 26.36) | 6.84 (1.48, 31.50) | 1 (ref) | 3.39 (0.67, 17.10) | 2.77 (0.53, 14.35) |

^a^ Adjusted for parental educational level, smoking during pregnancy, and child care

^b^ Median was 0.49 wt% for n-3 LC-PUFA, 0.17 wt% for DHA, 0.04 wt% for EPA, 0.37 wt% for AA, 0.44 for DHA/AA ratio and 0.07 for EPA/DHA ratio
